# Loss of Nf1 and Ink4a/Arf Are Associated with Sex-Dependent Growth Differences in a Mouse Model of Embryonal Rhabdomyosarcoma

**DOI:** 10.3390/cimb45020080

**Published:** 2023-02-02

**Authors:** Wade R. Gutierrez, Jeffrey D. Rytlewski, Amanda Scherer, Grace A. Roughton, Nina C. Carnevale, Krisha Y. Vyas, Gavin R. McGivney, Qierra R. Brockman, Vickie Knepper-Adrian, Rebecca D. Dodd

**Affiliations:** 1 Cancer Biology Graduate Program, University of Iowa, Iowa City, IA 52242, USA; 2 Medical Scientist Training Program, University of Iowa, Iowa City, IA 52242, USA; 3 Holden Comprehensive Cancer Center, University of Iowa, Iowa City, IA 52242, USA; 4 Department of Internal Medicine, University of Iowa, Iowa City, IA 52242, USA; 5 Department of Molecular Physiology and Biophysics, University of Iowa, Iowa City, IA 52242, USA; 6 Molecular Medicine Graduate Program, University of Iowa, Iowa City, IA 52242, USA

**Keywords:** rhabdomyosarcoma, sarcoma, cancer, mouse model, Nf1, Ink4a/Arf, vincristine

## Abstract

Rhabdomyosarcoma (RMS) is an aggressive form of cancer that accounts for half of all pediatric soft tissue sarcomas. Little progress has been made in improving survival outcomes over the past three decades. Mouse models of rhabdomyosarcoma are a critical component of translational research aimed at understanding tumor biology and developing new, improved therapies. Though several models exist, many common mutations found in human rhabdomyosarcoma tumors remain unmodeled and understudied. This study describes a new model of embryonal rhabdomyosarcoma driven by the loss of Nf1 and Ink4a/Arf, two mutations commonly found in patient tumors. We find that this new model is histologically similar to other previously-published rhabdomyosarcoma models, although it substantially differs in the time required for tumor onset and in tumor growth kinetics. We also observe unique sex-dependent phenotypes in both primary and newly-developed orthotopic syngeneic allograft tumors that are not present in previous models. Using in vitro and in vivo studies, we examined the response to vincristine, a component of the standard-of-care chemotherapy for RMS. The findings from this study provide valuable insight into a new mouse model of rhabdomyosarcoma that addresses an ongoing need for patient-relevant animal models to further translational research.

## 1. Introduction

Rhabdomyosarcoma (RMS) is an aggressive and deadly malignancy that accounts for half of all pediatric soft tissue sarcoma diagnoses annually [1]. The majority of these tumors develop in children younger than 10 years old, with an incidence of 4.71 per million in individuals less than 20 years old [2,3]. Rhabdomyosarcomas develop from skeletal muscle stem cells called satellite cells and can arise throughout the body, including in the limbs, trunk, head and neck, and pelvic regions. Tumors present as an expanding lump or swelling and may be painful, depending on the area of development. The exact cause of RMS development is unknown. However, genetic risk factors include the presence of cancer predisposition syndromes such as Li-Fraumeni syndrome, Neurofibromatosis type 1, Costello syndrome, Noonan syndrome, Beckwith-Wiedman syndrome, and DICER1 syndrome [4]. Unfortunately, the prognosis for childhood RMS has not substantially improved since the mid-1990s [5]. The prognosis for metastatic disease, which accounts for 15% of all new diagnoses, remains grim, with a five-year survival rate of only 31% [6]. Furthermore, chemotherapies such as vincristine (VCR) that are used to treat local and metastatic disease are fraught with side effects including persistent and debilitating neuropathy.

There are two main classes of pediatric RMS: alveolar RMS (ARMS), which comprises approximately 25% of cases; and embryonal RMS (ERMS), which comprises 60% of cases [2]. Though both arise from skeletal muscle tissue, ARMS and ERMS have substantially different genetic alterations, incidences, and outcomes, and they are characterized by distinct transcriptomic, epigenetic, and proteomic signatures [7]. ARMS frequently contains characteristic PAX3-FOXO1 or PAX7-FOXO1 fusion proteins and is associated with a worse prognosis than ERMS. Mouse models of ARMS similarly contain fusion genes such as Pax3-FKHR, with or without accompanying loss of tumor suppressors such as Ink4a/Arf or Trp53 [8,9]. In contrast, ERMS is fusion-negative and contains a wide variety of mutations. Over 50% of ERMS contain alterations in RAS pathway members, including NRAS, KRAS, HRAS, NF1, and FGFR4, as well as mutations in tumor suppressor genes such as TP53 and CDKN2A (INK4A/ARF) [10]. Mouse models of ERMS contain combinations of activating mutations in proto oncogenes such as HGF/SF, HER2-neu, and Kras and inactivating mutations in tumor suppressors such as Ink4a/Arf or Trp53 [11,12,13,14,15,16,17]. Though much work has been done developing RMS mouse models, many common mutations and unique combinations of mutations found in human ERMS tumors remain unmodeled and understudied [9,13,18,19,20,21,22]. The heterogeneity of genetic alterations in ERMS necessitates the development of new ERMS mouse models containing patient-relevant mutations to better understand tumor biology and rigorously test promising therapeutic regimens.

In this study, we developed and tested a new genetically engineered mouse model (GEMM) of ERMS driven by the loss of Nf1 and Ink4a/Arf. Tumors are generated by CreER technology, with CreER expression under the control of the satellite cell specific Pax7 promoter (Pax7^CreER/+^; Nf1^fl/fl^; Ink4a/Arf^fl/fl^, hereafter referred to as P7NI mice). Mutations in Nf1 and Ink4a/Arf are commonly found in ERMS tumors (15% and 4%, respectively), and this genetic combination follows the familiar pattern of Ras-pathway mutation coupled with the loss of a tumor suppressor that is found in other commonly-used RMS models [10,23,24]. To the best of our knowledge, this is the first report of an ERMS GEMM containing these alterations. We compared this new model to a well-established ERMS GEMM that relies on satellite cell-specific activation of oncogenic Kras^G12D^ and loss of the tumor suppressor Trp53 to drive tumor formation (Pax7^CreER/+^; Kras^LSL-G12D/+^; Trp53^fl/fl^) [13,14,15,16,17]. This preclinical GEMM, here referred to as P7KP mice robustly models histologic and molecular features of human ERMS tumors. We also describe two new murine ERMS cell lines and their corresponding immune-competent allografts which can be used for future translational studies of this aggressive cancer. Furthermore, we evaluate the efficacy of vincristine, a mainstay of pediatric RMS chemotherapy regimens, on cell lines and orthotopic allografts in multiple ERMS models. Taken together, this study provides new insight into the impact of initiating mutations on ERMS tumor biology.

## 2. Materials and Methods

### 2.1. Mice

All animal experiments were performed in accordance with protocols approved by the Institutional Animal Care and Use Committee at the University of Iowa. Mouse strains were maintained in the Dodd Lab colony in the University of Iowa Office of Animal Care barrier facilities. Same sex littermates were housed in standard shoebox housing (maximum of five animals per cage) with paper bedding and fed the facility’s standard irradiated mouse diet. Food and filtered drinking water were provided ad libitum. All cages and bedding were autoclaved before use. Animal health and welfare were assessed daily by veterinary staff throughout all phases of the experiment (breeding, tumor development, tumor growth). Animals were maintained on 12 h light/dark cycles and temperature and humidity-controlled rooms. Mice were maintained behind a barrier and monitored for contaminants by monthly sentinel harvest. The Pax7^CreER/+^; Kras^LSL-G12D/+^; Trp53^fl/fl^ (P7KP) sarcoma model has been previously described and is on a 129Sv/Jae background [13,15,17]. The Pax7^CreER/+^; Nf1^fl/fl^; Ink4a/Arf^fl/fl^ (P7NI) sarcoma model was generated by crossing previously-described Nf1^fl/fl^; Ink4a/Arf^fl/fl^ mice (C57Bl/6 background) [25] with Pax7^CreER/+^ mice (129Sv/Jae background) [26]. Following F1 generation, Pax7^CreER/+^; Nf1^fl/+^; Ink4a/Arf^fl/+^ mice were crossed with Nf1^fl/fl^; Ink4a/Arf^fl/fl^ mice to obtain Pax7^CreER/+^; Nf1^fl/fl^; Ink4a/Arf^fl/fl^ mice. These F2 mice were bred together for at least 3 generations before obtaining experimental mice used for this study. Experimental litters usually contain 4–6 pups, with approximately half of the mice containing the Pax7^CreER/+^ allele. To induce tumors in P7KP and P7NI mice, animals were injected with 50 µL of 4-hydroxytamoxifen (10 mM in DMSO, Sigma-Aldrich, St. Louis, MO, USA, H7904) in the gastrocnemius muscle. The gastrocnemius muscle is a commonly used site for tumor initiation and allows for easy and accurate assessment of tumor volume with digital calipers [13,15,17,27]. Mice were monitored a minimum of three times weekly to assess for general welfare and tumor initiation until all mice had developed tumors (final tumor development at 26 weeks post-tamoxifen injection). Following tumor initiation, tumors were measured three times weekly by a digital caliper. Tumor volume was calculated using the formula V = (π × *L* × *W* × *H*)/6, with *L*, *W*, and *H* representing the length, width, and height of the tumor in mm, respectively. Tumors were considered to have initiated when they reached 200–285 mm^3^ (approximately 7.5 mm × 7.5 mm × 7.5 mm). After tumors reached a volume of 1500 mm^3^ (approximately 14.2 mm × 14.2 mm × 14.2 mm), the mice were euthanized. In accordance with our approved animal protocol, mice were euthanized before tumors reached the maximum approved tumor volume of 2000 mm^3^. Mice were euthanized using both CO_2_ asphyxiation (primary method) and cervical dislocation (secondary method). A regional autopsy of the tumor and surrounding tissue was performed and samples of tumor tissue were collected. Male and female mice between 7 and 52 weeks old were used in all studies. P7KP: 12 male mice, 12 female mice. P7NI: 16 male mice, 12 female mice.

### 2.2. Histological Analysis

As previously described [17], terminal tumor tissue was stored in a 10% neutral buffered formalin for fixation and subsequent paraffin embedment. Formalin-fixed paraffin-embedded tumors were sectioned and stained with hematoxylin (Vector Laboratories, Newark, CA, USA, H-3401) and eosin (Harleco, Darmstadt, Germany, 200-12) to evaluate tissue morphology. Images were taken using an inverted microscope (Fisherbrand, Waltham, MA, USA, 03-000-013) with a digital camera attachment (Amscope, Irvine, CA, USA, MU503) at 40× magnification. Scale bars: 50 µm.

### 2.3. Derivation of Cell Lines

As previously described [17], terminal tumor tissue was dissected with forceps and surgical scissors, washed in 5 mL PBS in a 6-well plate, then finely minced with surgical scissors. Five mL of Dissociation Buffer [Collagenase Type IV (700 units/mL, Gibco, Waltham, MA, USA, 17104-019) and dispase (65 mg/mL, Gibco, Waltham, MA, USA, 17105-041)] in PBS was added to each well. Plates were incubated for one hour at 37 °C on an orbital shaker and transferred to a tissue culture hood. Dissociated tissue was passed through a sterile 70 µM cell strainer (Fisherbrand, Waltham, MA, USA, 22363548) into a 50 mL conical vial using a 10 mL serological pipette and the plunger from a 1 mL syringe (Becton Dickinson, Franklin Lakes, NJ, USA, 309628). Cell strainers were washed with 25 mL sterile PBS into corresponding conical vials. Cell suspensions were centrifuged, and cell pellets were resuspended and plated in DMEM (Gibco, Waltham, MA, USA, 11965-092). Cells were grown in 10 cm dishes maintained in DMEM media containing 10% FBS, 1% penicillin–streptomycin (Gibco, Waltham, MA, USA, 15140-122), and 1% sodium pyruvate (Gibco, Waltham, MA, USA, 11360-070). When 90% confluency was reached, 15–35% of cells were passaged into a new dish. Cell line morphology was monitored by microscope and lines were passaged a minimum of 10 passages (range 39–46 passages) until a consistent growth rate was achieved and no stromal cells were observed in the culture. Established cell lines were frozen for use in subsequent analysis. KRIMS-3 cells were derived from an untreated P7KP primary tumor, while NIMS-1 and NIMS-2 cells were derived from untreated P7NI primary tumors.

### 2.4. Generation of Orthotopic Syngeneic Allografts

As previously described [17], cells were ~90% confluent on the day of injection. Cells were trypsinized, washed, and resuspended in sterile PBS containing calcium chloride and magnesium chloride. To develop orthotopic allografts, mice were injected with 50 µL of cell suspension (4 × 10^5^ cells total) in the left gastrocnemius muscle using a 31G needle. For P7KP allografts, KRIMS-3 cells were injected into mice maintained on a KP (Kras^LSL-G12D/+^; Trp53^fl/fl^) background. For P7NI allografts, NIMS-1 and NIMS-2 cells were injected into mice maintained on a P7NI background. Same sex littermates were maintained in the Dodd Lab colony in the University of Iowa Office of Animal Care barrier facilities. Animals were housed in standard shoebox housing (maximum of five animals per cage) with paper bedding and fed the facility’s standard irradiated mouse diet. Food and filtered drinking water were provided ad libitum. All cages and bedding were autoclaved before use. Animal health and welfare were assessed daily by veterinary staff throughout all phases of the experiment (breeding, tumor development, and tumor growth). Animals were maintained on 12 h light/dark cycles and temperature and humidity-controlled rooms. Mice were monitored a minimum of three times weekly to assess tumor initiation. Mice that had not developed tumors by 35 days after the injection of cells were euthanized. Following tumor initiation, tumors were measured three times weekly by a digital caliper. Tumors were considered to have initiated when they reached 200–285 mm^3^ (approximately 7.5 mm × 7.5 mm × 7.5 mm). On the day of tumor initiation, mice were randomized to receive one dose of vincristine (Selleckchem, Houston, TX, USA, S1241) diluted in water or saline (PBS) via intraperitoneal injection. Randomization occurred by an alternating enrollment scheme. Animals were all housed on the same rack, and cage location was not changed during the study. Subsequent tumor growth measurements were collected in a blinded manner. After tumors reached a volume of 1500 mm^3^ (approximately 14.2 mm × 14.2 mm × 14.2 mm), mice were euthanized. In accordance with our approved animal protocol, mice were euthanized before tumors reached the maximum approved tumor volume of 2000 mm^3^. In cases of tumor regression, mice were euthanized 35 days after tumor initiation. Mice were euthanized using both CO_2_ asphyxiation (primary method) and cervical dislocation (secondary method). A regional autopsy of the tumor and surrounding tissue was performed and samples of tumor tissue were collected. Male and female mice between 7 and 52 weeks old were used in all studies. KRIMS-3 allografts: 4 male mice, 8 female mice. NIMS-1 allografts: 12 male mice, 9 female mice. NIMS-2 allografts: 13 male mice, 10 female mice.

### 2.5. Cell Growth and Vincristine Sensitivity Assays

As previously described [17,27], KRIMS-3, NIMS-1, and NIMS-2 cells were grown in 10 cm dishes maintained in DMEM media containing 10% FBS, 1% penicillin-streptomycin (Pen-Strep, Gibco, Waltham, MA, USA, 15140-122), and 1 mM sodium pyruvate (Gibco, Waltham, MA, USA, 11360-070). For cell growth assays, cells were plated on Day 0 in a 12-well plate (2.5 × 10^4^ cells). On Day 1, resazurin (Sigma-Aldrich, St. Louis, MO, USA, R7017) dissolved in PBS (1.5 mg/mL) was added to wells (200 µL for 12-well) and cells were returned to the tissue culture incubator for 1–2 h before being read on a microplate reader (BioTek, Winooski, VT, USA). This process was repeated 24 h later on Day 2. Fluorescence measurements from Day 2 were normalized to measurements from Day 1 for each cell line to calculate the 24 h fold growth. For vincristine dose response assays, cells were plated on Day 0 in a 96-well plate (KRIMS-3: 8.8 × 10^3^ cells, KRIMS-4: 9.2 × 10^3^ cells, and NIMS-1: 8 × 10^3^ cells, NIMS-2: 5 × 10^3^ cells). On Day 1, vincristine (Selleckchem, Houston, TX, USA, S1241) diluted in water was added at varying concentrations to wells. On Day 2, viability was assessed using a resazurin assay (20 µL per well) as described above. Fluorescence values were normalized to wells containing 0 µM vincristine. Dose-response curves were fitted using nonlinear regression, option “[inhibitor] vs. normalized response-variable slope” in GraphPad Prism (Version 9, Boston, MA, USA).

### 2.6. Statistics

Statistical analysis was performed using GraphPad Prism (Version 9, Boston, MA, USA) 8. In vivo, time-to-tumor and survival curves were analyzed using a Log-rank (Mantel-Cox) test with Bonferroni correction (if comparing more than two groups). In vitro data were analyzed using Welch’s ANOVA and Dunnett’s T3 multiple comparison tests.

## 3. Results

### 3.1. Tumor-Initiating Mutations Impact Rms Development and Growth

**Figure 1:**
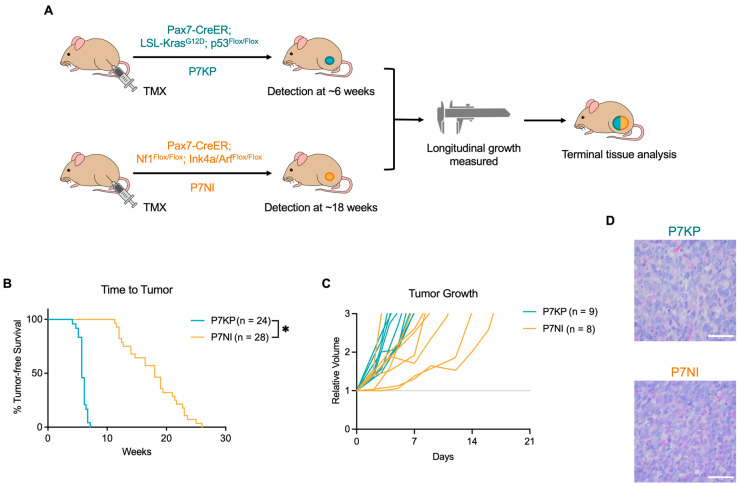
P7KP and P7NI tumor initiation, growth, and histology. (**A**) P7KP and P7NI tumors were induced using intramuscular injection of tamoxifen (TMX) to locally delete Trp53 and activate oncogenic Kras^G12D^ (P7KP) or to locally delete Nf1 and Ink4a/Arf (P7NI). Following tumor initiation, tumor dimensions were measured by caliper three times weekly and terminal tumor tissue was collected for analysis. (**B**) P7NI tumors initiated significantly slower than P7KP tumors. (**C**) P7NI tumors also displayed a broader range of growth rates than P7KP tumors. (**D**) P7KP and P7NI tumors share similar histologic features including cells with high cytologic variability and eosinophilic cytoplasms. Scale bar: 50 μM. Log-rank (Mantel-Cox) tests used to analyze B. * *p* < 0.05.

Both RMS models used in this study develop tumors from muscle satellite cells, which functioned as the stem cells of skeletal muscle. CreER expression was under the control of the satellite cell-specific Pax7 promoter. Under normal conditions, CreER remains in the cytoplasm. However, in the presence of tamoxifen (TMX), CreER translocates to the nucleus, allowing for Cre recombinase activity at LoxP sites. In P7KP mice (Pax7^CreER/+^; Kras^LSL-G12D/+^; Trp53^fl/fl^), intramuscular injection of TMX caused expression of oncogeneic Kras^G12D^ and a loss of Trp53 and satellite cells, resulting in RMS formation [13,14,15,16,17]. Similarly, in P7NI mice (Pax7^CreER/+^; Nf1^fl/fl^; Ink4a/Arf^fl/fl^), intramuscular injection of tamoxifen led to the biallelic loss of Nf1 and Ink4a/Arf in satellite cells at the site of injection (Figure 1A). P7KP and P7NI tumors are histologically similar, with both models displaying the histopathologic hallmarks of RMS, including high cytologic variability and small round cells with eosinophilic cytoplasms and hypochromic nuclei (Figure 1D). However, though they share the same cell of origin, the difference in genetic mutations has a profound impact on tumor initiation and growth. While all animals developed ERMS tumors following TMX injection (Figure 1B), tumor latency was significantly different between the two models. Similar to previous reports [17], P7KP tumors (n = 24) initiated between 4.1 and 7.1 weeks (median 5.7 weeks) after TMX injection (Figure 1B). P7NI tumor onset (n = 28) was significantly slower, requiring 11.3 to 26.0 weeks (median 18.0 weeks) to initiate following TMX injection. Similarly, the growth kinetics of the tumors were also genotype-specific. While P7KP tumors tripled in volume within six days (range of four to seven days) after tumor initiation (Figure 1C), P7NI tumors displayed a more varied range of growth patterns, tripling in volume in nine days (range 3 to 17 days). Indeed, the majority of P7NI tumors (six of eight) grew slower than the slowest P7KP tumor which tripled in volume in seven days.

### 3.2. RMS Cell Lines Are Sensitive to Vincristine Treatment In Vitro

**Figure 2:**
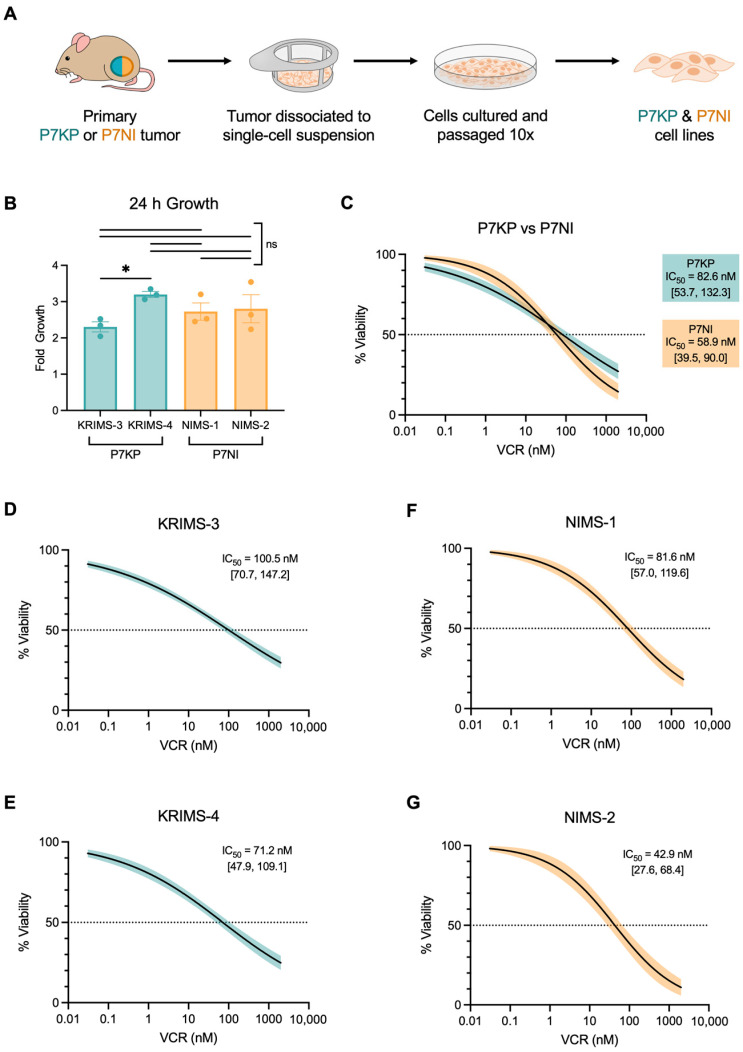
Development and characterization of P7KP and P7NI cell lines. (**A**) Terminal P7KP and P7NI tumors were enzymatically dissociated and cultured to develop cell lines. KRIMS (Kras Induced Murine Sarcoma) cells were derived from P7KP tumors and NIMS (Nf1 Induced Murine Sarcoma) cells were derived from P7NI tumors. (**B**) KRIMS and NIMS growth in vitro. Cells were seeded at equal densities and growth was assessed after 24 h. Data points represent independent experiments (n = 3). (**C**–**G**) Vincristine (VCR) dose-response curves. Cells were treated for 24 h. (**C**) Average dose-response curves by genotype. (**D**–**G**) VCR dose-response curves for individual cell lines. Data from 3–4 independent experiments included in each plot. Curves fitted using nonlinear regression, option “[inhibitor] vs. normalized response-variable slope” in GraphPad Prism (Version 9, Boston, MA, USA). Shaded areas represent 95% confidence intervals. Absolute IC_50_ 95% confidence intervals displayed in brackets. Welch’s ANOVA and Dunnett’s T3 multiple comparison test used to analyze B. * *p* < 0.05; ns = not significant.

To further assess the impact of initiating mutations on tumor biology, we developed cell lines from primary RMS tumors (Figure 2A). Terminal tumor tissue was enzymatically dissociated and cultured in vitro. Cell lines were passaged a minimum of 10 times to facilitate the removal of nontumor stromal cells. We have previously published findings with two P7KP cell lines that we developed using this method, known as Kras Induced Murine Sarcoma 3 and 4 (KRIMS-3 and KRIMS-4 cells) [17]. In the current study, we derived two new P7NI cell lines called Nf1 Induced Murine Sarcoma 1 and 2 (NIMS-1 and NIMS-2 cells).

In vitro growth assays demonstrated that both KRIMS and NIMS cells grow at very similar rates (Figure 2B). As previously reported, KRIMS-4 cells grow slightly faster than KRIMS-3 cells (3.2-fold versus 2.3-fold increase after 24 h). In contrast, there is no difference in growth between NIMS-1 and NIMS-2 cells. In addition to evaluating growth rates, we assessed the sensitivity of each cell line to vincristine (VCR), a microtubule inhibitor commonly included in the multidrug chemotherapy regimen used to treat RMS (Figure 2C–G). Dose-response studies determined that IC_50_ values for P7KP cells and P7NI cells were both in the midnanomolar range, including an average of 82.6 nM for KRIMS cells (KRIMS-3: 100.5 nM and KRIMS-4: 71.2 nM) and 58.9 nM for NIMS cells (NIMS-1: 81.6 nM and NIMS-2: 42.9 nM) (Figure 2D–G). Taken together, these data demonstrate that cells from the P7NI model closely resemble cells from previously-published P7KP models in vitro.

### 3.3. P7NI Tumor Growth Phenotypes Are Sex Dependent

**Figure 3:**
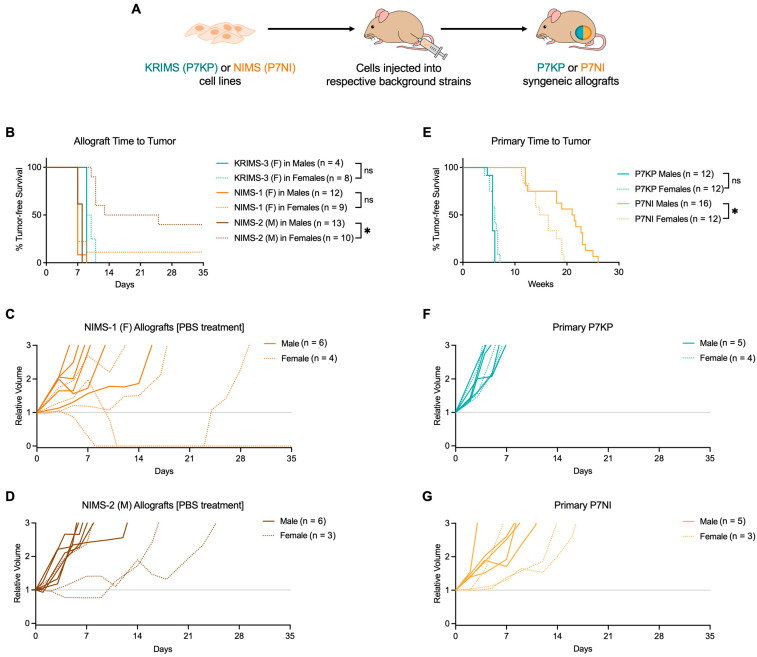
KRIMS and NIMS allograft development and growth. (**A**) KRIMS (P7KP) and NIMS (P7NI) cells were orthotopically implanted into mice of the same respective background strain to develop syngeneic allografts. KRIMS-3 and NIMS-1 cells were derived from tumors in female (**F**) mice, while NIMS-2 cells were derived from a tumor in a male (M) mouse. (**B**) NIMS-2 cells displayed significant sex-dependent differences in tumor initiation. (**C**,**D**) Individual NIMS-1 and NIMS-2 tumor growth curves. Tumors in females developed later than those in male mice and displayed highly variable growth patterns. (**E**) Reanalysis of data presented in Figure 1B. Tumors in female P7NI mice initiated significantly faster than in male P7NI mice. (**F**,**G**) Reanalysis of data from Figure 1C. Individual primary P7KP and P7NI tumor growth curves. Log-rank (Mantel-Cox) test with Bonferroni correction used to analyze B (adjusted α = 0.003125) and E (adjusted α = 0.008333). * *p* < 0.003125 in B; * *p* < 0.008333 in E. Complete statistical analysis available in Figure S1. * *p* < 0.05; ns = not significant.

One major goal of our study was to test the sensitivity of RMS mouse models to vincristine (VCR), an integral member of the multidrug standard-of-care chemotherapy for RMS patients. To facilitate translational studies of Nf1-deleted RMS in vivo, we developed orthotopic syngeneic orthotopic allografts with the NIMS cell lines (Figure 3A). Allografts offer several advantages to primary genetically engineered tumors, including more rapid and consistent tumor initiation. Upon tumor initiation, mice were randomized to receive either vincristine (VCR) or saline control (PBS). We compared the two new NIMS allografts to the well-established KRIMS-3 allografts [17] (Figure 3B and Figure S1A, Table 1). As previously reported, KRIMS-3 allografts arise nine to eleven days following cell implantation and show similar tumor initiation trends in male and female mice. Given the similarities between KRIMS and NIMS cells in vitro, we hypothesized that NIMS allografts would follow a similar pattern of initiation and growth. To our surprise, we observed significant sex-dependent differences in tumor growth of the NIMS allografts. In males, all mice implanted with NIMS-1 and NIMS-2 cells developed tumors within seven to nine days following cell implantation. In contrast, tumor development was substantially lower in female mice, with only six of ten female mice developing NIMS-2 tumors and seven of eight female mice developing NIMS-1 tumors. Of note, the differences between the NIMS-1 and NIMS-2 allograft initiation rates in female mice may be due to the sex of the mice from which the cell lines were derived, as NIMS-1 cells were developed from a female mouse while NIMS-2 cells were developed from a male mouse. However, this would be a NIMS-specific characteristic, as both of the previously published KRIMS-3 and KRIMS-4 cell lines derived from female mice develop allografts equally in male and female recipient mice (Figure S1B) [17].

In accordance with our original experimental design, mice were randomized to treatment with saline (PBS) or VCR at the time of tumor initiation. In PBS-treated Nf1-deleted tumors, we observed sex-dependent tumor growth patterns (Figure 3C–D, Table 1). In male mice, NIMS-1 and NIMS-2 allograft growth patterns were similar to primary P7NI tumors. However, in female mice, NIMS-1 allografts displayed inconsistent growth trajectories. Surprisingly, two NIMS-1 tumors in female mice regressed to unmeasurable levels. One of these tumors became detectable again after 14 days (24 days after initial cell implantation), while the other remained stably regressed at five weeks post cell implantation. Though none of the PBS-treated NIMS-2 allografts in female mice regressed to unmeasurable levels, two of the three tumors displayed substantially slower growth than the NIMS-2 allografts in male mice. Based on these findings, we reanalyzed tumor initiation data from the primary tumor models presented in Figure 1B,C. As previously reported [17], onset of P7KP primary tumors did not differ based on sex (n = 12 males, n = 12 females, Figure 3E and Figure S1C). In contrast, primary P7NI tumors in female mice initiated significantly faster than in male mice (median 15.6 weeks for the 12 female mice versus 21.2 weeks for the 16 male mice, respectively) (Figure 3E and Figure S1C). Similarly, longitudinal growth curves of primary P7NI tumors resembled those of PBS-treated NIMS allografts, with tumors in females trending toward slower growth than those in males (Figure 3G). P7KP primary tumors, in contrast, showed no growth differences between male and female mice (Figure 3F).

### 3.4. Both P7KP and P7NI Allografts Are Resistant to Vincristine

**Figure 4:**
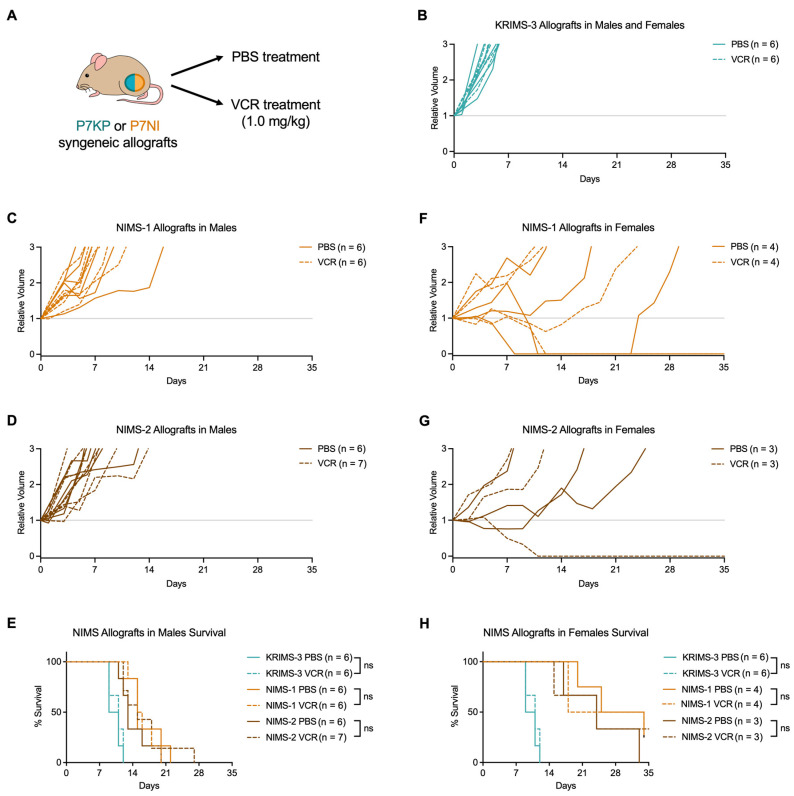
Impact of vincristine on allograft growth. (**A**) Mice were treated with one dose of saline (PBS) or vincristine (VCR) at the time of tumor initiation. (**B**) Individual growth curves for KRIMS-3 allografts in male and female mice. (**C**,**D**) Individual growth curves for NIMS-1 and NIMS-2 allografts in male mice. (**E**) Survival of NIMS-1 and NIMS-2 allografts in male mice and KRIMS-3 allografts in male and female mice. (**F**,**G**) Individual growth curves for NIMS-1 and NIMS-2 allografts in female mice. (**H**) Survival of NIMS-1 and NIMS-2 allografts in female mice and KRIMS-3 allografts (same KRIMS-3 allografts shown in **D**). Log-rank (Mantel-Cox) test used to analyze sets of PBS versus VCR treated in mice E and H. ns = not significant.

For KRIMS allografts, the impact of VCR treatment was assessed using combined groups of males and females since sex affects neither the P7KP primary tumor nor KRIMS-3 allograft initiation or growth (Figure 3E,F, Figure 4A,B and Figure S1B). Given the substantial sex-dependent differences in NIMS allograft tumor initiation and growth, we assessed the impact of VCR treatment on males and females separately (Figure 4C–H). A single dose of VCR (1.0 mg/kg) was administered at the time of tumor initiation.

This dose was chosen since it is close to the maximum-tolerated dose in a previous study reporting that doses of VCR greater than 1.0 mg/kg in mice are associated with total body weight loss greater than 10% and death (LD_50_ = 3.8 mg/kg) [28]. We found that at this dose, VCR treatment did not impact KRIMS-3 allograft growth (Figure 4B) or survival (Figure 4E). Likewise, VCR did not affect the growth or survival of NIMS-1 and NIMS-2 allografts in male mice (Figure 4C–E). In female mice with NIMS-1 allografts, VCR had no appreciable effect, although one mouse treated with VCR experienced complete and sustained tumor regression, similar to one of the PBS-treated female NIMS-1 allograft mice described above (Figure 4F, Table 1). Similarly, in VCR-treated female mice with NIMS-2 allografts, one mouse experienced complete and sustained regression. However, this effect is not generalizable, as two VCR-treated NIMS-2 tumors in female mice showed rapid growth. Importantly, VCR did not extend survival in female mice with either NIMS-1 or NIMS-2 allografts (Figure 4H). Taken together, these data indicate that VCR has a minimal impact on tumor growth and survival in both P7KP and P7NI orthotopic syngeneic allografts, suggesting these tumors are models of chemotherapy-resistant rhabdomyosarcoma.

## 4. Discussion

The heterogeneity of genetic alterations in RMS necessitates the development of new mouse models to better understand RMS biology and to test promising therapeutic regimens more rigorously. In this study, we have described a new mouse model of ERMS driven by the loss of Nf1 and Ink4a/Arf in muscle satellite cells (P7NI mice). To assess the characteristics of the model, we compared it to a previously validated murine ERMS model that relies on activation of oncogenic Kras^G12D^ and a loss of Trp53 (P7KP mice). We found that the primary P7NI tumors had delayed onset and grew more slowly than P7KP tumors. Cell lines derived from P7KP and P7NI tumors (KRIMS and NIMS, respectively) behaved similarly in vitro, but orthotopic syngeneic allografts generated from these cells did not follow this pattern. Unexpectedly, NIMS allografts displayed sex-dependent differences in tumor initiation and growth that were not present in KRIMS allografts. These findings prompted us to reanalyze data from the primary genetically engineered tumors. We found that the onset of P7NI tumors was five weeks earlier in female mice than in male mice. To better understand the utility of the P7NI models in preclinical studies, we assessed the sensitivity of cell lines and orthotopic syngeneic allografts to the microtubule inhibitor VCR. We found that in vitro P7KP and P7NI cell lines were equally sensitive to VCR. However, in vivo studies in orthotopic syngeneic allografts demonstrated that neither genotype was responsive to 1.0 mg/kg VCR treatment, a previously-reported maximum tolerated dose [28]. These immune-competent preclinical models of drug-resistant RMS will be a valuable tool to better understand the mechanisms underlying resistance and to identify new means of overcoming resistance.

As previously stated, to the best of our knowledge, this is the first animal model of NF1-mutated RMS, which accounts for 15% of all ERMS tumors [10]. In human ERMS, the impact of individual mutations on tumorigenesis and the age of diagnosis is not well-documented. However, mutations in TP53 have been associated with worse outcomes (shortened event-free survival times) [10]. The preservation of Trp53 in P7NI mice may partially explain the slowed tumorigenesis and tumor growth in this model compared to P7KP tumors. Other ERMS genetic signatures associated with decreased overall survival and failure-free survival include increased expression of EPHA2 (ephrin type-A receptor 2), NSMF (NMDA receptor synaptonuclear signaling and neuronal migration factor), and EPB41L4B (erythrocyte membrane protein band 4.1 like 4b) [29]. The impact of these genetic alterations on the response to specific treatments such as vincristine is not well understood due to the variable rates of genetic testing and regional differences in treatment regimens. Mouse models of ERMS, such as those described in this study, are critical for furthering our understanding of the impact of genetic changes on ERMS biology and response to treatment.

We also used this model to explore RMS response to vincristine in vitro and in vivo. Importantly, RMS patients are commonly treated with a multidrug chemotherapy regimen comprised of vincristine, actinomycin D, and cyclophosphamide (termed VAC). We chose to focus our studies on vincristine monotherapy for several reasons. First, we wanted to directly compare responses between in vitro and in vivo models. Since cyclophosphamide is a prodrug and requires exogenous activation to work in tissue culture, it is difficult to properly control its activity experimentally. Second, we were concerned about the cumulative toxicities of the VAC regimen and instead chose to use vincristine alone at the published maximum tolerated dose. Our data determined that both the P7KP and P7NI models are resistant to vincristine in vivo. Both intrinsic and acquired resistance to chemotherapy is common occurrences in human RMS. Recently, the upregulation of the Zinc finger protein GLI1 was identified in vincristine-resistant RMS and Ewings Sarcoma cell lines [30]. Other groups developed a series of 34 RMS xenografts to evaluate intrinsic and acquired chemotherapy resistance to the VAC regimen [31]. Studies such as ours describing intrinsically resistant immune-competent RMS models complement this previous work on acquired resistance using in vitro systems and immune-deficient xenografts.

The P7NI model uniquely models sex-dependent differences in tumor initiation and growth. Of note, ERMS incidence is slightly higher in males compared to females [2], although this male predominance is also seen in the majority of other childhood cancers [1,32]. However, in cohorts of pediatric patients, rates of NF1-mutated cancers are significantly higher in females than in males [33,34]. Though most of these differences were in cancers originating in the central nervous system such as optic pathway gliomas, it was possible that a similar Nf1-associated mechanism was promoting the earlier tumorigenesis in female P7NI mice. Of note, very few studies have examined sex-dependent differences in NF1-mutated tumor initiation and growth. Though studies of NF1-mutated tumors in human patients typically contain both males and females, data from the two sexes are often combined, likely due to limited cohort sizes. Additional studies are needed to better understand the impact of sex on NF1-mutated tumor biology in human and in animal models.

The newly described P7NI model has both strengths and limitations. The strengths discussed above include the unique genetics of the model, the ability to examine chemotherapy resistance, and the sexual dimorphism of tumor growth. A limitation of the primary GEMM P7NI model is slow tumor onset (median time 18 weeks) and a wide range of tumor initiation times. These factors make treatment studies in the primary model cumbersome. Though their derivative allografts had much shorter times to tumor initiation, they also displayed inconsistent initiation rates in female mice. These strengths and limitations should be taken into account when considering the use of P7NI primary or allograft tumors in experimental designs. Future studies with this model could examine the in vivo response to the VAC chemotherapy regimen and compare these outcomes to vincristine monotherapy data. Similarly, this model could be used to test targeted therapies in tumors with different initiating mutations.

In conclusion, we have demonstrated that a new mouse model of ERMS driven by the loss of Nf1 and Ink4a/Arf histologically resembles previously-published ERMS models while also displaying unique features, including delayed tumor initiation and sex-dependent growth patterns. The new P7NI primary tumor model, cell lines, and orthotopic syngeneic allografts will be useful tools in furthering our understanding of the impact of genetic heterogeneity on RMS biology. Additionally, this model may be of use in evaluating mechanisms underlying intrinsic resistance to VCR. Further investigation is needed to better understand the mechanisms driving observed sex-dependent differences in tumor growth.

## Figures and Tables

**Table 1:** KRIMS and NIMS allograft development, growth, and treatment.

		**KRIMS-3 (F)**		
Sex	Initiated	Regressed	Reached 3×	Treatment
M	Yes	No	Yes	PBS
M	Yes	No	Yes	PBS
M	Yes	No	Yes	VCR
M	Yes	No	Yes	VCR
M Total	4/4	0/4	4/4	2:2
F	Yes	No	Yes	PBS
F	Yes	No	Yes	PBS
F	Yes	No	Yes	PBS
F	Yes	No	Yes	PBS
F	Yes	No	Yes	VCR
F	Yes	No	Yes	VCR
F	Yes	No	Yes	VCR
F	Yes	No	Yes	VCR
F Total	8/8	0/8	8/8	4:4
		**NIMS-1 (F)**		
Sex	Initiated	Regressed	Reached 3×	Treatment
M	Yes	No	Yes	PBS
M	Yes	No	Yes	PBS
M	Yes	No	Yes	PBS
M	Yes	No	Yes	PBS
M	Yes	No	Yes	PBS
M	Yes	No	Yes	PBS
M	Yes	No	Yes	VCR
M	Yes	No	Yes	VCR
M	Yes	No	Yes	VCR
M	Yes	No	Yes	VCR
M	Yes	No	Yes	VCR
M	Yes	No	Yes	VCR
M Total	12/12	0/12	12/12	6:6
F	Yes	No	Yes	PBS
F	Yes	No	Yes	PBS
F	Yes	**Yes**	Yes	PBS
F	Yes	**Yes**	**No**	PBS
F	Yes	No	Yes	VCR
F	Yes	No	Yes	VCR
F	Yes	No	Yes	VCR
F	Yes	**Yes**	**No**	VCR
F	**No**	-	-	-
F Total	8/9	3/8	6/8	4:4
		**NIMS-2 (M)**		
Sex	Initiated	Regressed	Reached 3×	Treatment
M	Yes	No	Yes	PBS
M	Yes	No	Yes	PBS
M	Yes	No	Yes	PBS
M	Yes	No	Yes	PBS
M	Yes	No	Yes	PBS
M	Yes	No	Yes	PBS
M	Yes	No	Yes	VCR
M	Yes	No	Yes	VCR
M	Yes	No	Yes	VCR
M	Yes	No	Yes	VCR
M	Yes	No	Yes	VCR
M	Yes	No	Yes	VCR
M	Yes	No	Yes	VCR
M Total	13/13	0/13	13/13	6:7
F	Yes	No	Yes	PBS
F	Yes	No	Yes	PBS
F	Yes	No	Yes	PBS
F	Yes	No	Yes	VCR
F	Yes	No	Yes	VCR
F	Yes	**Yes**	**No**	VCR
F	**No**	-	-	-
F	**No**	-	-	-
F	**No**	-	-	-
F	**No**	-	-	-
F Total	6/10	1/6	5/6	3:3

## Data Availability

The data presented in this study are available on request from the corresponding author.

## Supplementary Materials

The following supporting information can be downloaded at: https://www.mdpi.com/article/10.3390/cimb45020080/s1, Figure S1: Statistical analyses and KRIMS-3 tumor growth.
