# Supplementary material for: Loss of Nf1 and Ink4a/Arf Are Associated with Sex-Dependent Growth Differences in a Mouse Model of Embryonal Rhabdomyosarcoma
Source: Curr Issues Mol Biol. 2023 Feb 2;45(2):1218–32. doi: 10.3390/cimb45020080 (PMC9955904; doi:10.3390/cimb45020080)
Supplement: Supplementary file 1 [file cimb-45-00080-s001.zip › Pax7 Supplemental Figures 221101.pdf]

A

		KRIMS-3		NIMS-1		NIMS-2	
		M	F	M	F	M	F
KRIMS-3	M		ns	*	ns	*	*
	F	ns		*	ns	*	*
NIMS-1	M	*	*		ns	ns	*
	F	ns	ns	ns		ns	*
NIMS-2	M	*	*	ns	ns		*
	F	*	*	*	*	*	

B

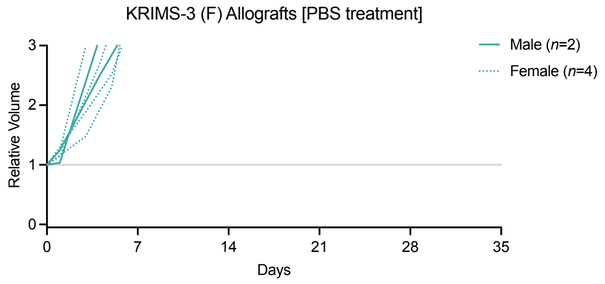

C

	P7KP M	P7KP F	P7NI M	P7NI F
P7KP M		ns	*	*
P7KP F	ns		*	*
P7NI M	*	*		*
P7NI F	*	*	*	

Supplemental Figure 1: Statistical analyses and KRIMS-3 tumor growth. (A) Statistical analysis for Figure 3B. (B) Growth of KRIMS-3 allografts in male and female mice. (C) Statistical analysis for Figure 3E. Log-rank (Mantel-Cox) test with Bonferroni correction used to analyze A (adjusted α = 0.003125) and C (adjusted α = 0.008333). *$P < 0.003125$ in A; *$P < 0.008333$ in C.
